# Marijuana, *feijoada* and the debate on drug legalization

**DOI:** 10.3389/fpsyt.2013.00007

**Published:** 2013-03-05

**Authors:** José A. S. Crippa, Jaime E. C. Hallak, Antonio W. Zuardi

**Affiliations:** ^1^Departamento de Neurociências e Ciências do Comportamento da Faculdade de Medicina de Ribeirão Preto da University of São PauloRibeirão Preto, Brazil; ^2^Instituto Nacional de Ciência e Tecnologia, Translacional em Medicina, CNPqRibeirão Preto, Brazil

*Feijoada* is one of the most typical dishes of Brazilian cuisine, commonly made from a mixture of black beans and several cuts of pork and beef. It is served with rice, *farofa* (toasted, seasoned manioc flour), sautéed collard greens, and sliced oranges, among other side orders. In the most sophisticated recipes it can take more than 30 ingredients, including spices and side dishes. One does not have to be a cook, chef, or expert to distinguish beans—the main ingredient of the dish—from complete *feijoada*.

Marijuana, the popular name of *Cannabis sativa*, has more than 400 compounds, many of which are named “cannabinoids” (substances that affect receptors carrying the same name) (Pate, 2002). In an analogy with *feijoada*, Δ9-tetrahydrocannabinol (Δ9-THC), responsible for the psychoactive effects of the drug (Zuardi, 2008), could be the beans.

In the early twentieth century, when the active principles of the drug had not yet been isolated, marijuana extracts were marketed by large pharmaceutical companies for a number of indications (Fankhauser, 2002). However, the therapeutic use declined within a few years due to the difficulty in obtaining reproducible effects and to the introduction of other drugs for the same indications of marijuana at that moment. Marijuana extracts have a wide variability in their composition, stability, and potency. Thus, consonant with the principles of the evolution of pharmacotherapy, effort has been put into the development of purer cannabinoid compounds that can be accurately measured, thus reducing the risk of significant undesirable side effects.

In the first half of the 1960s the chemical structures of the major cannabinoids were determined by Professor Raphael Mechoulam from Israel, including Δ9-THC. Around 80 cannabinoids have been described to date, with different effects and many with therapeutic potential.

Cannabidiol (CBD), for example, which makes up to 40% of marijuana extracts, has several effects opposite to those of Δ9-THC, including anxiolytic and antipsychotic properties (Crippa et al., 2010). Differently from Δ9-THC, the use of CBD alone does not cause the typical effects of marijuana, and Brazilian, American, British, and Israeli groups are in the leading edge of research on the therapeutic potential of this compound (Carlini, 2010). Today, CBD is being tested in Brazil in Parkinson's disease, schizophrenia, social phobia, post-traumatic stress disorder, smoking, epilepsy, depression, and other conditions (Crippa et al., 2010; Schier et al., 2012).

It can be said, therefore, that cannabinoids are components of marijuana, but that the two are not synonyms. That is, cannabinoids are not marijuana.

Marijuana is the most commonly used illicit drug in many countries, despite evidence showing that it may cause transient psychiatric symptoms and cognitive alterations depending on the dose. Moreover, chronic marijuana use may also cause long-lasting cognitive alterations and trigger the onset of psychiatric disorders in vulnerable individuals, depending on the dose, frequency, and earliness of use (Solowij et al., 2002; Manrique-Garcia et al., 2012), although these findings are still under debate.

The marijuana withdrawal syndrome has gained increased recognition and it is known that some individuals may develop dependence (Hasin et al., 2008). Currently available therapeutic interventions—both pharmacological and non-pharmacological—have shown less than optimal efficacy. Modern neuroimaging studies show alterations in brain function with chronic, repeated use of marijuana (Bhattacharyya et al., 2012). Furthermore, clinical complications such as cancer and breathing and immunological problems have also been associated with the use of the drug. However, as cannabis is often smoked in conjunction with tobacco and/or other drugs, the relationship between chronic use and these problems is so far inconclusive (Lader, 2009).

Therefore, the effects of chronic marijuana use on health need careful evaluation. The existing evidence about these effects is also confounded and misleading, as it considers marijuana and cannabinoids to be equivalent.

All scientific debate concerning the legalization of marijuana should necessarily be informed by empirical data from clinical trials and epidemiological studies. Any debate occurring on a background of political positions, ideological biases and, even worse, personal beliefs will only increase confusion, and postpone concrete decisions.

Cannabinoids and drugs that act in the endocannabinoid system have been shown to have a fantastic therapeutic potential and there is reason to believe that they could benefit millions of people worldwide. A better understanding of the mechanisms of action of these compounds, with the ensuing legalization of cannabinoids, would be an outstanding scientific breakthrough, leading to a significant decrease in burden, and improved quality of life for people with many diseases and disorders. Conversely, the debate on the legalization of marijuana for recreational purposes should only take place after society and the scientific community is clearly informed about the potential complications of the drug or its possible low-risk profile. In order to do this, however, it is crucial to separate the wheat from the chaff, beans from *feijoada* or—in this case—cannabinoids from marijuana.
